# Supplementary material for: Sexual and reproductive health needs of women with severe mental illness in low- and middle-income countries: A scoping review
Source: PLoS One. 2025 Jan 16;20(1):e0311554. doi: 10.1371/journal.pone.0311554 (PMC11737715; doi:10.1371/journal.pone.0311554)
Supplement: Attachment — Submitted filename: PLOSONE_Scoping review.docx [file pone.0311554.s004.docx]

Thank you for allowing me to review the paper, “Sexual and Reproductive Health Needs of Women with Severe Mental Illness in Low and Middle-Income Countries: A Scoping Review”. This is an important and timely review which clearly lays out the existing research in this area and the gaps. I found this review very well written and the methods and results clearly articulated. I only have a few comments/suggestions.

Background

1. It would be helpful to include a definition of what is meant by SMI within the background section.

Methods:

1. The inclusion criteria is clear. However, it may also be helpful to include specific exclusion criteria applied.
2. I can understand a potential rationale for not having a restriction on date of publication, but it would be helpful to outline why no restriction on date was chosen.
3. It states there was no restriction on language. How were papers not published in English screened?
4. It may be helpful to reference the full example of a search that is currently in the supporting information in the text. Additionally, it would be good to reference that the checklist is also in the supporting information.
5. I found Figure 3 confusing and difficult to interpret. For example, the difference in number of studies between Egypt and Peru is difficult to see. Is there another way to present this data? Maybe by including the *n* in the circles? You could even just use a bar chart showing frequency of publications for each country?

Results

1. For the findings on the lack of reporting of ethical procedure, could this be explained by changes in norms of reporting ethical procedures throughout the years? For example, is this information mostly missing from papers published from over 10 years ago? If not, it is still interesting to present this data to show that even in recent publications this information is missing.
2. How were the six themes identified? I have seen more formal methods liked thematic synthesis or thematic analysis used to help identify themes for scoping reviews? Was a specific method used? Please explain what method/process was used and why? For example, were these identified themes agreed upon by the whole team?
3. In discussion of the themes, you could include some specific examples/more details from some of the included studies, especially those that include qualitative data to get a better sense of how the papers are discussing the identified needs in their specific contexts. Now the themes are very descriptive which help answer your first research question around extent and type of evidence. I would hope that some of the themes could be developed further to be more interpretive to help understand/summarise the needs identified (the last part of your research aim).
   - For example, Pg 25 lines 364-365, you give some information about a specific study, but I would want to know a little more here. For example, what were the main perceptions and experiences of mental health care staff? As is done with thematic synthesis, you could even pull out some specific quotes from the paper.

Discussion

1. I wonder if the information in “Comparison to Previous Literature” could be moved to the background section?
2. Pg 31, “Study type will need careful consideration as many of the studies included in 508 this review were observational and may provide rich insights but be challenging to draw conclusions from”. I think now there are some mixed-method systematic review methodologies that would help address this so I don’t think it is true that it would be challenging to draw any conclusions from these types of studies.

Thank you once again for all your work on this review. It was great to read and nicely lays out the next steps and areas in need of further research and development.
